# Brain talk: power and negotiation in children’s discourse about self, brain and behaviour

**DOI:** 10.1111/j.1467-9566.2012.01531.x

**Published:** 2012-10-25

**Authors:** Ilina Singh

**Affiliations:** Department of Social Science, Health and MedicineKing's College, London

**Keywords:** ADHD, brain, self, children, Ritalin

## Abstract

This article examines children’s discourse about self, brain and behaviour, focusing on the dynamics of power, knowledge and responsibility articulated by children. The empirical data discussed in this article are drawn from the study of Voices on Identity, Childhood, Ethics and Stimulants, which included interviews with 151 US and UK children, a subset of whom had a diagnosis of attention deficit/hyperactivity disorder. Despite their contact with psychiatric explanations and psychotropic drugs for their behaviour, children’s discursive engagements with the brain show significant evidence of agency and negotiated responsibility. These engagements suggest the limitations of current concepts that describe a collapse of the self into the brain in an age of neurocentrism. Empirical investigation is needed in order to develop agent-centred conceptual and theoretical frameworks that describe and evaluate the harms and benefits of treating children with psychotropic drugs and other brain-based technologies.

## Introduction

In recent years an increasing neurologisation of the person has been emerging not just as an ontological concern but also, in the social science literature, as an empirical reality. For social scientists the new era of neurocentrism is being driven by the modern individual’s interaction both with neurotechnologies themselves (such as functional magnetic resonance imaging machines, psychotropic medications or brain stimulation devices) and with the discursive power of neuroscientific ‘facts’ that proliferate in modern life, particularly in the media. As a consequence, it is argued, we have come to think of the ‘brainome’ as the new blueprint for personal identity and ethical self-stylisation (Rose 2007). We read essential dimensions of the self – mood, personality, cognition, morality and gender – through the brain, primarily via neurochemistry and brain scan images. Our subjectivity has been colonised by the neuro (Broer and Heerings 2012), and we are reduced to ‘cerebral subjects’ (Vidal 2009).

If we have been cerebral subjects for quite some time (Vidal 2009) it is all the more surprising that a critique of sociology’s own romance with the brain has only recently emerged. A series of recent articles have shown both the empirical limitations of key theoretical concepts such as the ‘neurochemical self’ (Rose 2007) and the extent to which sociological accounts may be involved in co-constructing the problem of neurocentrism itself (Pickersgill *et al.* 2011). Focus group and internet-based research among individuals with different mental illness diagnoses as well as among healthy adults and adolescents suggest that assumptions about the importance of neuroscience or brain-based explanations of identity, mental health and illness, and development may need to be fundamentally rethought (Broer and Heerings 2012, Choudhury and Ortega 2011, Pickersgill *et al.* 2011). The brain may not, in fact, always be a privileged site of subjectivity, nor are its inner workings necessarily associated with key dimensions of the self. Moreover, brain-based explanations, or excuses for behaviour that does not fit popular conceptions of normality, may be used instrumentally and unevenly in groups and by individuals (Martin 2007, Singh 2004, 2005). The accumulating empirical evidence suggests that, at least in Anglo-American communities, individuals resist significant external pressures to subjugate the self to the brain.

Children have fared particularly poorly in a time of co-constructed neurocentrism. Neuroscience research can reduce social, moral, cognitive and emotional development to brain correlates, and is used to justify social and educational interventions that target the brain and promise to grow children up to be productive citizens (Foresight Report 2008). At the same time, sociological accounts often reduce children to passive victims of medicalisation and neuroscience technologies such as psychotropic drugs (see Singh 2011 for a review). Such accounts persist despite the fact that the broader field of social studies of childhood and child health has, for over a decade now, been moving away from social theories that define children as passive or docile in the face of social and medical forces. A compelling body of work has focused on children’s capacity for agency and has positioned children as key social actors whose voices should and do make a unique contribution to social worlds (for example, Bluebond-Langner and Korbin 2007, James 2007, Mayall 1998, Thorne 2006). This work reconfigures the theoretical association between childhood and vulnerability that tends to produce representations of the child as victim. As Bluebond-Langner and Korbin (2007) point out, children are among the most vulnerable members of society; however, along with the concept of childhood itself, children’s vulnerabilities are variable and context-dependent rather than universal or determined. To disregard children’s capacity for agency, even as part of a justified critique of the structural constraints imposed on children, misses an important opportunity to discover resilience as well as vulnerability in children’s experiences of health-related practices, interventions and discourses.

One of the most compelling correctives to the currently limited sociological perspective on children and psychotropic drugs is to be found in sociological work on children and non-medical technologies, specifically media technologies. Here, sociologists have drawn on empirical social science research to reveal the sentimentality and essentialism embedded in the assumption that the digital media age represents the death of childhood (Buckingham 2000, Prout 2005, Turkle 1997). Prout (2005) suggests that this sentimentality about the natural or innocent state of the child is rooted in a false nature–culture dualism. The notion that children are victims of technologies or of the reductive narratives that often accompany technologies elides the fact that ‘the entities we call “biological”, “technological” and “social” are already networked together’ (Prout 2005: 141). Because the sense of an ‘I’– of a persisting self – emerges within this network rather than outside it, over time a child is likely to integrate new technologies with a personal identity rather than to feel the loss of a pre-technological childhood she never knew.

The figure of a networked, emergent, bio-techno-social actor represents a critical move to situate children’s subjectivities in a different relation to technological developments. This article extends these efforts to build a complex, agent-centred account of children, health, technology and childhood to research on children’s engagements with neuroscience discourse and psychotropic drug treatments. I also hope to contribute to the emerging critique of co-constructed neurocentrism in the sociology of psychiatry and neuroscience. Despite these broad aims, my analytic focus is narrow: drawing on data from an international study on children’s experiences with attention deficit hyperactivity disorder (ADHD) diagnosis and stimulant medications, I examine children’s discursive constructions of the relationship between self, brain and behaviour. My emphasis here is not the narrative emplotment of the brain, as in Martin’s (2007) elegant brain stories, but rather the dynamics of power, knowledge and responsibility that reveal themselves in a discursive self-brain-behaviour triangle. Because I am interested in how a self is constructed and expressed through this triangle (which I take to represent a version of a bio-techno-social network), I refer not to an objective third person entity that is the self, but to the first person entity-under-construction: the ‘I’. My argument is that, despite contact with psychiatric explanations and technologies for their behaviour, children’s discursive engagements with the brain show significant evidence of agency and independent decision-making. At the end of the article I briefly contextualise these engagements in parent and peer accounts of the relation between self, brain and behaviour. Finally, I suggest the need for more empirical investigations that help us better understand where children are resilient and where they are vulnerable, in relation to technologies that intervene on the brain.

## Methods

This article draws on data collected from the study, Voices on Identity, Childhood, Ethics and Stimulants (VOICES).^1^ The study used a mixed-methods approach to examine the social and ethical implications of ADHD diagnosis and stimulant drug treatments, paying particular attention to issues of personal agency, authenticity, stigma and responsibility. We recruited 151 families in the US and the UK between 2008 and 2010. The child participants were between the ages of 9 and 14. Approximately 70 per cent of the children we interviewed were boys. Most participants in the study were White and lower middle-class. There were three groups of children in each country: (A) children diagnosed with ADHD and taking stimulant medication; (B) children diagnosed with ADHD and not medicated and (C) children without a psychiatric diagnosis. We found that in the real world these categories were not mutually exclusive, in that some children who were recruited for the second group – diagnosed but unmedicated – had previously taken medication for ADHD. We recruited children for the undiagnosed group using a behavioural screen that ensured that this group exhibited a similar range of positive and negative behaviour to children with a diagnosis of ADHD. Parents and children completed standardised forms and measures in addition to the interview. VOICES received NHS ethics approval in the UK and institutional research board approval in each US site where we recruited patients.

### Interview structure and analysis

Children did not just start talking to us about their brains. We prompted them to talk about their brains in relation to their behaviour at two different points in a one hour interview that included semi-structured and structured questions, standardised pictures, drawing, sentence completion tasks, an ordering task and a vignette. Our discussions about the brain, personal identity and behaviour with children invited them to articulate their conception of the brain’s role in behaviour and in personal identity, but left a lot of room for them to propose alternative influences. Children with experience of medication and diagnosis spoke specifically about those experiences in relation to identity, brain and behaviour. Children without these direct experiences were asked to imagine behavioural scenarios and to discuss if and how these related to the brain. For example, all the children were asked to recount a recent time when they had felt really good about their behaviour and a recent time when they had felt very bad about their behaviour. They were then asked: ‘Do you think your brain has anything to do with how you behave?’ Children without a diagnosis were also asked to discuss the behaviour of children with ADHD (if they knew any) and to consider if and how this behaviour might relate to those children’s brains. Because the brain was an unusual topic for most children, those sections of the interview moved from semi-structured questions to more specific, structured questions. Most children engaged willingly in these sections of the interview.

It is notable how few children reported specifically having learned about the brain or aspects of neuroscience in school. Older children’s understanding or knowledge of the brain was not markedly different from that of younger children. Most children referred to how brains were represented in cartoons or stories; television programmes were also common sources of knowledge. Children occasionally mentioned that parents or doctors had referred to the brain in their explanation to children of their difficulties. Despite these reports of a lack of learned knowledge, children’s discussion about the brain frequently tapped into popular representations of how the brain works; for example, as an electrical circuit board or via localised functions. Therefore children’s understandings about the brain were substantively intersected with a ‘folk neurology’ (Vrecko 2006), even though children generally could not pinpoint specific sites where they had learned particular pieces of information.

The children’s interviews in the VOICES study were subjected to several iterations of qualitative analysis as well as quantitative content analysis (see also Singh 2011). Careful construction of the topic guide enabled focused analyses in different sections of the interview. Initially a systematic thematic analysis of all interviews was conducted using a coding frame that had been validated by a coding team. Case studies were created to examine and to illustrate the conceptual value of the ecological niche (Bronfenbrenner 1979) as a way to describe the proximal and distal influences on a child’s development. In the course of analysis it became evident that the thematic analysis was not sufficiently granular to capture the interesting dynamics of power, responsibility and decision-making that children articulated as part of the relationship between self, brain and behaviour. Therefore, relevant sections of interviews (sections where children responded to questions about their brains) were subsequently re-analysed by the author using discourse analysis, with high level themes serving as a conceptual foundation. To make the data analysis manageable, groups (A), (B) and (C) in each country were first stratified into two age groups; 9–11 and 12–14. Six interviews that were deemed unusable for this analysis (because children did not engage sufficiently with the relevant interview questions) were set aside. Within each of the age-stratified groups, interviews were randomly sampled until saturation of discursive patterns was achieved.^2^ This resulted in a sample of 67 interviews. Once this sample had been analysed, one further interview from each age-stratified group was randomly chosen to check for consistency of the findings. Using this approach it was possible to more carefully explore children’s discourse about the ‘I’, agency, relationality and power in the thematic areas, revealing the subtle discursive variations described in this article. It should be noted that this sampling process inevitably introduced some bias; and, while an effort was made to ensure reliability of findings within the dataset, a discourse analysis on a different sample from this data may lead to somewhat different conclusions.

Two ecological niches were found in the VOICES study – a ‘performance niche’ and a ‘conduct niche’ (see also Singh 2011, 2012). Briefly, in a performance niche, children’s cognitive achievements and successes are strongly emphasised by children and adults, ADHD is viewed as a disorder of academic performance or learning and children are likely to report effects of stimulant drugs in relation to classroom behaviour, school work, intelligence and academic achievements. In a conduct niche, children’s social behaviour and social hierarchies are a dominant preoccupation among children and adults, and academic achievement has similar or less value than other obligations. ADHD is more likely to be viewed as a problem of anger and aggression, and stimulant drugs tend to be seen by children as targeting emotional self-control, aggressive behaviour and decision-making.

In other articles on the VOICES data, the role of the ecological niche that children inhabit is foregrounded because of its importance in differentiating children’s experiences of ADHD diagnosis and stimulant drug treatments. However, in the analysis performed for this article, few qualitatively significant differences were found in children’s articulation of ‘I’-brain-behaviour dynamics at the level of the ecological niche or, indeed, at the level of children’s diagnosis or treatment status.^3^ Therefore the analytic decision was taken to foreground discourse patterns in ‘I’-brain-behaviour negotiations and to fill in surrounding contextual factors as relevant. As a consequence of this decision, this article does not present a focused investigation of the local values and expectations that shape children’s knowledge and discourses about self, brain and behaviour, although a preliminary effort is made to contextualise children’s discourse in parental beliefs and peer influences.

## Negotiating with my brain

In light of concerns outlined above about the relegation of subjectivity to the brain, it is relevant to ask what sort of status children give the brain in relation to the self and behaviour, and relatedly, how they see the brain working – what sort of power does it have? In the VOICES interviews, children articulated a dynamic model of ongoing negotiations between the self and the brain, with consequences for behaviour. These negotiations were characterised by changing attributions of power (here defined as the ability to influence behaviour) to the self and to the brain, such that there was a continuum of shifting power relations rather than any one hegemonic power expressed across negotiations. In what follows I describe how children articulate the shifting power relations between the self and the brain, and how they view the resulting impacts on behaviour.

### The weakest ‘I’

Whether or not children had a diagnosed behavioural disorder, most could identify situations in which they felt they did not have sufficient control over impulsive behaviour or the degree to which they paid attention or listened to an adult. These experiences led some children to hypothesise that the brain must be a powerful source of control over their behaviour. This is exemplified in statements such as ‘your brain controls everything; without your brain you couldn’t do anything; or without your brain, you’d be dead’. In this depiction, many children see the brain as fundamentally instrumental: it ‘helps me pick up a pen and stuff like that’ or ‘it moves my arms and legs or it makes me breathe’.

However, even in the articulation of this powerful brain, subjectivity is not collapsed into the brain. There is an observing ‘I’ present. This ‘I’ is separate from the brain, although it is weak, particularly in face of the brain’s instrumental power. This weak ‘I’ is most visible, discursively, when children feel that the brain is using its power to negative ends (this is usually when children have done something bad, for which they hold the brain responsible). Then there is a virtuous struggle, between the brain and a weak ‘I’:

The brain is like probably like, the king and queen of the body. And so it’s telling the body what to do. It can do whatever it wants. Like, if you just had a fight with your best friend and like, said bad things. Or like you spread a rumor about someone … Then you like say, like, I wish I could go back and change that. That’s when your brain’s acting for you … It’s like the brain has its own brain that has the ADD. (Angie, US, age 11, previously on medication for ADHD)

In Angie’s vision, even a very powerful brain has its unruly dimensions, which can cause trouble. Children conceive of these unruly dimensions in various ways. Angie imagines that ADHD has its own control module within the brain and occasionally manages to take the brain over. The result is behaviour that Angie feels is not morally right. Observing the behaviour of her body from a distance, Angie’s ‘I’ is present but too weak to do anything but wish it could change the things the brain made it do: ‘I wish I could go back and change that’.

### A vulnerable brain

Angie’s ‘I’ is the weakest and rarest form of subjectivity that can be consistently identified in children’s narratives: an ‘I’ that is present and observing but helpless to overrule the actions dictated by a powerful (but still vulnerable) brain. Angie names the brain’s unruly dimension ‘ADD’. More commonly, children describe the brain’s vulnerabilities (which include ADHD but are not exclusively caused by ADHD) in less concrete terms, as problems characterised by disconnection – either among brain parts or among the brain, the ‘I’ and behavioural outcomes. Children describe this as parts of the brain that are not connecting up, or are sending bad signals, or receiving no signal, or are lighting up, working too hard or on fire. As a result, the brain is ‘out of wack’ or off balance. In Zach’s words:

Well, there’s like, there’s like two wires there and, um, connected to the brain, and, um, they’re not – they’re like – and then there’s like, you know, how normally they’re connected together, like the whole brain, like a series of wires and like a circuit board. It’s all connected. If you didn’t have one wire working, that section of the thing doesn’t work so if they’re connected … the light bulb won’t light up… The brain’s not working. That section of the brain’s not working so it’s like, telling my brain to mess around and be stupid – say stuff that’s not relevant. (Zach, UK, age 14, taking medication for ADHD)

Contextual factors can exacerbate or cause these brain troubles. Children in a performance niche, who feel more academic stress, report that their brains ‘work too fast’ or are ‘full of noise’. Children in a conduct niche, who experience regular bullying and aggression, frequently describe their brains as overheated or explosive. Children in both niches sometimes associate physical pain with these brain processes. In a performance niche the pain is likely to be in or around the brain; for example, a headache. In a conduct niche the pain is also likely to be narrated through the body:

Usually my heart goes doof, doof … After I’ve been running for a while, if I lie down I can see my heart going against my chest … Usually if I do get too angry … I’d just feel my brain like that [makes pounding noise] or something … I don’t know. It does hurt my eyes when it’s bad. (Timothy, UK, age 11, taking medication for ADHD)

Amid this noise, heat, pain and disconnected confusion, a weak ‘I’ stands little chance of making good decisions about the right behaviour in a given context. Children report how challenging it is to negotiate the brain’s internal confusion and vulnerabilities:

[S]ometimes your brain might tell you to do something and you want to do it, but you don’t want to do it, but you do it anyway and you end up getting into trouble. (Sharon, US, age 10, diagnosed with ADHD, not on medication)

Nevertheless, many children express a forceful subjectivity in the power dynamics between the brain and the ‘I’. The first step in self-assertion is to be clear that the brain is an unreliable source of information about normative behaviour. As Justin explains:

Justin: Usually it [the brain] tells me what it’s the right time to be my jumpy self and the wrong time. And sometimes it tells me the wrong thing, like when I should be calm, I’m hyper.

INT: Why do you think it does that?

Justin: Well, because it wants to be hyper and thinks it’s the right time. (Justin, US, age 14, taking medication for ADHD)

Given the brain’s unreliability, the ‘I’ that wants to avoid ‘getting into trouble’ needs to be able to perform a series of complex tasks. It must be able to listen to an incoming message from the brain, recognise the problematics of its source, evaluate the rightness or wrongness of a message in light of context and choose the right action. As Justin notes, these tasks are made more difficult by a dangerous equality between the brain and the ‘I’: the brain can be as stubborn about what it wants to do as the ‘I’– and as wrong in its estimation of appropriate behaviour.

Such personification of the brain occurs frequently in children’s accounts. In contrast to occupying a reified status as transcendent authority, the brain can exhibit vulnerabilities that mimic the child’s own difficulties. However, personification of the brain is not the same as personalisation; in other words, the brain’s childlike vulnerabilities are not cast as part of a narrative in which a child’s subjectivity merges with the brain. Instead, the brain’s vulnerabilities can potentiate a child’s agency. Although critics of psychiatric diagnosis may rightly balk at some of the ‘problems’ children identify with their brains (such as ‘I have too much imagination’), these perceived problems do in many cases motivate a creative young-folk neurology that demonstrates children’s perception of a shared authority between the brain and the ‘I’:

I suppose, when you’re in a bad mood, some people say, you don’t think straight or you just focus on one thing, but I don’t think your brain could, like, calm you down. It’s up to you to do that, not your, it’s not to do with your brain … Um, sometimes when you’re hyper and like that, then your heart beats really fast and if you just sit down and relax, and your heart slows down, you know, and you become a bit calmer. (Constance, US, age 11, no diagnosis)

In its most powerful form, a knowing, observing and independent ‘I’ emerges in children’s narratives, whose role is to protect the ‘I’ and the brain from the brain’s vulnerabilities. To do this, the ‘I’ needs to achieve executive control. As Vaughn explains:

INT: Does your brain control your behaviour?

Vaughn: Myself. I’m in control.

INT: So what does your brain do then?

Vaughn: My brain gives me an idea and I decide if I should do that. (Vaughn, US, age 9, previously on medication for ADHD)

Part of the wisdom of this ‘I’ is its recognition of the brain’s powerful influence on behaviour, as well as the brain’s capacity to resist the authority of the ‘I’. Therefore negotiations need to be managed firmly:

Your brain, it’s like actually what you do. Like you have to tell your brain that you’re not going to do that; you have to be strong to your brain. You can’t just like let it win.(Laurence, UK, age 10, taking medication for ADHD)

### A disconnected ‘I’

At the same time (and sometimes in the same interview) children express a different power dynamic between the ‘I’ and the brain in their negotiations over behaviour. The brain can also be a positive influence, in which case, the agency exhibited by the ‘I’ is more the problem:

Graham: Basically my brain controls, it basically controls my whole body.

INT: Is there anything your brain doesn’t control?

Graham: Um, when I listen and such.

INT: Do you think that it’s your brain’s not working properly?

Graham: Mm, no, I think it’s like I probably don’t choose to listen.(Graham, US, age 10, diagnosed with ADHD, not taking medication)

In depicting himself as ‘choosing’ not to listen (in contrast to saying he can’t listen), Graham refuses a role in which he is the victim of some defect and instead highlights the ‘I’’s investment in ongoing negotiations with the brain. In keeping with this investment, children tend to describe the ‘I’’s inability to listen to the brain not as a problem of a defective brain or of a defective ‘I’, but as a problem of relationship. Not listening to a brain that is trying to tell the ‘I’ to avoid problematic behaviour may be a sign that the ‘I’ is wilfully disconnected from the brain:

Sometimes when [my brain is telling me to stop] I don’t, I really don’t listen or I just want to keep going or I tell myself it’s ok, it’s ok, you can keep doing this … Maybe, it’s just, like I can’t connect with what I’m, with what my brain is trying to tell me, and I want to do something else, and I really just don’t care. (Jodi, US, age 12, taking medication for ADHD)

In this interaction the ‘I’ faces its own vulnerabilities (not listening, not caring) that drive the disconnection with the brain and the resulting behaviour. The ‘I’’s agency here is again notable; it is not depicted as a helpless victim of defects. Rather, it is a desiring, striving, stubborn actor whose brain has to work to keep up in a series of ongoing relational power struggles.

The fact of these ongoing dynamics between the ‘I’ and the brain ultimately means that the ‘I’ usually has a choice: to listen to the brain or not. Sometimes the brain is telling the ‘I’ the right thing to do, and sometimes it is not. But the ‘I’ maintains decision-making power and final responsibility for behavioural outcomes. In the following excerpt Oscar struggles to explain this process of negotiation and the power dynamics involved:

Your brain controls everything like around your body … But it can’t, it can’t like, control you. You control it, but it knows what you’re going to do, what you’re about to do. But you can control the brain. Like, you can do things that you want, like, your brain to let you do but … I mean, let’s say you have to do the back flip, but your brain is like, oh my gosh, don’t do it, don’t do it. Then you shouldn’t do it; it’s too risky. (Oscar, US, age 12, taking medication for ADHD)

In Oscar’s explanation, the brain functions as a sort of superego, or conscience, warning the ‘I’ about the risks of foolish behaviour. If conscience is related in some way to the capacity to reason (Arendt, 2005), then clearly there is something rather profound at stake in these negotiations. As Conrad reveals, children associate the ability to reason about actions, both before and after actions have taken place, with moral character:

Um being good and bad is to do with thinking about your actions. Um, and I think if you’re bad, then you don’t really think about your actions very much. You just, or you just, um, you do think about them; it’s just that you want to, um, get a laugh or a kick out of what you’re doing. (Conrad, UK, age 13, taking medication for ADHD)

Indeed, children with ADHD talk frequently about feeling that a major consequence of the disconnection between the ‘I’ and the brain is a reduced ability to reason – to stop and think before they act. The ‘I’ can feel too weak to manage this important job alone – or, the ‘I’ realises too late that the brain was sending a wrong message about how to behave, and the damage has already been done.

Stimulant medication plays an important role here, in that children feel it enables connection and listening between the ‘I’ and the brain, and helps the ‘I’ make good decisions. The effect of stimulant medication that children diagnosed with ADHD in this study most consistently value is its impact on their ability to make more space for reasoning and decision-making (Singh 2012). As Nicholas says in the extract below, children feel that with medication they have more decision-making power than before. The normative capacities of the ‘I’ are strengthened:

[Tablets] slow down my brain, I think, from being so hyper… my brain’s like too fast, it’s like ahead of everything. I just do things and sort of without any thoughts. [Medication] makes me think straight. (Nicholas, UK, age 14, taking medication for ADHD)

Medication, however, is not generally seen by children as a panacea, nor do children view medication as an exclusive solution to the problem of the disconnection between the ‘I’ and the brain. Although many children feel that medication is a helpful influence, they report that a better connection between the ‘I’ and the brain can also be fostered with the help of good friends, good teachers, music, quiet and physical activity. These influences ‘slow things down in my brain’, so that mutual listening between the ‘I’ and the brain is possible. In children’s reported experiences, such listening allows for better decision-making, which leads ultimately to more intentional (and less impulsive) behaviour:

Victoria: There’s like a fireball. It’s like bursting. It wants to get out and then it sort of like travels up to my head. And then … [makes exploding noise]. Sometimes I can control it and sometimes I just don’t … I like sort of squash it with like … I like to eat, so like I eat quite a lot … I have a meditation tape I sometimes use. It’s got three tracks and they’re different lengths … I like go outside and stuff like run, walk in the fresh air and stuff.

INT: And that prevents the fireball from coming out?

Victoria: Yes. But sometimes I don’t think. Sometimes it doesn’t help.(Victoria, UK, age 12, not taking medication)

A small minority of children espouse the radical belief that the disconnection between the ‘I’ and the brain is of a fundamental nature. As part of this belief, the brain is an instrumental power with which the ‘I’ has to negotiate. Other parts of the body, or transcendent entities, are the ‘I’’s existential home. Other parts of the body children mention are the heart and ‘nerves’. Transcendent entities include God and the soul. While this belief was not mentioned by many children (and this could have been a function of the way we asked our questions), it is nevertheless a further example of how children’s discourse refutes the notion that subjectivity is necessarily read through the brain:

Your brain is practically your battery. So if you was a robot, the brain would be your battery. It tells you when to shut down, when to move, how much to move, right, 30 degrees and all that stuff. And um, well, then there is a soul, and well um a soul is just who you are, what you are … Your soul is your soul. You can’t get rid of it; you’re stuck with it forever, until you die. (Don, UK, age 11, taking medication for ADHD)

## An embedded triangle: I-brain-behaviour negotiations in context

The preceding discussion illustrates that children interviewed in the VOICES study articulate a resilient sense of self in relation to the brain, even when they have been exposed to psychotropic drugs and psychiatric diagnosis. At the level of discourse, children do not tend to subjugate the ‘I’ or behaviour to brain-based explanatory models. Rather, children tend to describe ‘I’–brain relations that emphasise their capacity and desire for personal agency. Indeed, the creativity and intelligence in children’s imagined dialogues between the ‘I’ and the brain indicate that many children are able to ensure a distinct role and status for the ‘I’ in its ongoing negotiations with the brain.^4^

One question arising from this analysis relates to the wider influences that may shape children’s ‘I’-brain-behaviour negotiations. How do children’s ideas intersect with those of influential others in the ecological niche? A substantive discussion of this important question is beyond the scope of this article; however, two brief observations about these intersections can be made to provide some context for the finding of children’s resilient ‘I.’

The first observation concerns parents of children with ADHD. Many parents of children we interviewed overtly espoused brain-based explanatory models for their children’s behaviour. In brief conversations in front of their children with the interviewer present and on our standardised forms, parents made statements like, ‘I’m so glad to know that it’s a problem with his brain, not with him’ or, ‘Now I can tell his teacher it’s not his fault that he behaves the way he does’. Several mothers of children we interviewed had been treated for depression and anxiety, which they associated with the stress of managing a difficult child. The exculpatory ‘brain blame’ narrative was apparently a real relief to these mothers. How children retain agency in their negotiations with the brain in light of the surrounding reliance on brain blame rhetoric warrants further investigation. One possible explanation is found in the growing body of work on parents of children with ADHD and other conditions. A good deal of this research suggests that although parents will use a model of biological causation for rhetorical relief from parent blame (and mother blame especially) and for instrumental purposes (such as ensuring disability resources for a child), the model may not support actual relief from feelings of personal responsibility, anxiety, guilt and blame (Blum 2007, Callard *et al.* 2012, Hansen and Hansen 2006, Singh 2004, 2003). Nor does a model of biological causation necessarily have a consistent influence on parenting behaviour or decisions about treatments (Singh 2005). Children may therefore incorporate a more complex and conflicted set of beliefs about the causes of their difficulties, as these beliefs are embedded in parenting practices and the home environment, while they also learn to use the instrumental ‘excuse’ of biology when it is in their interest or when they know they can get away with it (Singh 2011, Singh *et al.* 2010).

A second observation concerns the undiagnosed peer group of children with ADHD. Children with ADHD are justifiably concerned about stigma relating to ADHD – particularly that children will make totalising assumptions about them based on the label and drug treatment. In our interviews children with and without a diagnosis told us: ‘ADHD means you’re mental; ADHD means you’re stupid; if you take Ritalin you’re a druggie’. Such descriptions classically serve to mark individuals with the diagnostic label as ‘other’ and to categorise their experiences as deviant from the norm (Goffman 1990). Interestingly, however, when undiagnosed children talk about their negotiations with their brains, they frequently articulate challenges similar to those articulated by children with a diagnosis. In the extended excerpt below, Arnie (UK, no diagnosis, age 9) sounds much like the children with ADHD to whom we have listened in this article:

INT: Do you think your brain has anything to do with how you behave?

Arnie: … Yeah, instead of doing something, like, you don’t want to do, like, if you’ve got most of your brain covered in, like badness, you would, um try [to do the bad thing], even though there’s only a little bit trying to make you good, you’re most likely to do, um, something bad.

INT: Let me just repeat to make sure I’ve got this right. So if this bit … is, you say, covered in badness [pointing to drawing child has made of the brain] … what happens?

Arnie: Um, it will most likely make you, um, um, do something that you want to do, but … um, you have a 50/50 chance, but if it’s like a quarter … if it’s only a quarter of badness, you’ll have, like, less chance of doing something you don’t want to do …

INT: So, you say it’s something you don’t want to do … Do you feel like you’re doing something you’re not supposed to do?

Arnie: Yeah, after, after when you actually do it, you feel like I didn’t want to do that … Because my friend, R, he’s short, and then I shouted at him and then he started crying because I went up to him because … I was bigger than him so then he started crying and ran home … I thought, when it stopped, I thought I’ve just done something I didn’t want to do.

The discussion with Arnie suggests that, contrary to what is implied by the stigmatising rhetoric around ADHD, the struggle to exercise self-control and the imagined moral dimensions of ‘I’-brain-behaviour negotiations, are not unique to children who have the diagnostic label. Arnie makes it clear that the embodied challenge to ‘stop and think’ before acting is part of children’s normal developmental experience. As such, we might speculate that the young folk neurology that grows out from these experiences of ‘I’-brain-behaviour negotiations resists reductive explanations for behaviour precisely because the experiences of struggle with self-control are common, rather than abnormal.

When they move beyond discussion of the label and consider what is different about children with ADHD, most undiagnosed children do not construct reductive or categorical evaluations of children with ADHD. Rather, undiagnosed children tend to think that children with ADHD face more formidable challenges than they do, in their (familiar) negotiations with the brain. Moreover, because the brain is not necessarily seen as the only authority or the most powerful influence, in ‘I’-brain-behaviour negotiations there is room for children to imagine non-brain-based solutions to the problem of behaviour. Arnie goes on to say:

Arnie: [Kids with ADHD] probably have that much goodness [shows on drawing of brain] because they have really bad problems, so, um, if they, if they, um, they’ll most likely get, um, upset and then start going mad … But if they don’t, then they’ll probably have this much badness [shows a smaller amount] and if they’re not bad at all because their mum and dad don’t let them, they’ll be perfectly fine.

INT: Do you think their mum and dad have something to do with it?

Arnie: Yeah, because of, um, how they act when they were children, um, could affect what, what’s happening to their children.

INT: … What happens if your parents, like, show you how to act and stuff, what would happen then?

Arnie: Um, the brain will actually kind of like this, if they keep on teaching you every day … then on the last day they teach you, it’ll probably have this much badness [shows on drawing] so all of this is good. They teach you. And then if they teach you a little bit more, you’ll be full.

As with parental influences on children’s negotiations of ‘I’-brain-behaviour power dynamics, it will be important to further investigate peer influences on children’s discourses, particularly with regard to peer influences on children with a diagnosed psychiatric disorder. There is as yet no substantial body of empirical work to inform hypotheses in this area. What seems clear is that children have a common experience of struggling with self-control, although the struggles are seen to be more acute in diagnosed children. The experience of struggle, when made explicit, can give rise to complex ideas about the relationship between the ‘I’, the brain and behaviour. Interestingly, these ideas seem to have more in common with contemporary scientific understanding of brain plasticity and less in common with concepts that position the brain as a hegemonic influence over the ‘I’ or over individual behaviour.

## Conclusion

Neuroscience discourse and neurotechnologies increasingly infuse our lives and the lives of children. Children currently encounter neuroscience knowledge and tools in their daily lives primarily through gaming technologies (for example, brain training games) and mainstream education, where neuroscientific knowledge is supporting an increasing emphasis on educating (training) the brain and on generating cognitive classifications of ability, potential and achievement (Foresight Report 2008, Howard-Jones 2007). A growing number of children around the world are introduced to neuro-discourses and tools more directly, via psychological or psychiatric treatments. Some psychiatric treatments have made their way out of the clinic and into the everyday experiences of young people because of their dual-use potential as cognitive enhancers. Between 10 and 15 per cent of US university students use psychotropic medications as ‘study aids’ (Singh and Kelleher 2010), and brain–computer interfaces are becoming available commercially to help individuals ‘achieve full mental potential’ (see Neurobit Systems (n.d.).

Such technologies will have varied impacts and meanings for children, depending on the technology in question, the ways in which it enters a child’s life and the surrounding social and political factors that mediate the child’s encounter with the technology. As this article has shown, it is not the case that encounters with neuroscience discourse or technologies necessarily lead children to construct neurological subjectivities. Rather, children are seen to be active and creative participants in discursive power negotiations among social, biological and technological forces. Throughout these negotiations many children sustain a resilient sense of self and agency, perhaps because the embodied experience of moral struggle over self-control outweighs reductive explanations for behaviour that they may hear from adults.

The discovery of children’s resilient subjectivity here supports the work of other scholars who have suggested the need for a more empirically grounded, conceptually nuanced sociology of neuroscience. It also supports a move away from theorising children as docile in face of norms and expectations that would shape their bodies, behaviour and cognition. When we acknowledge children’s vulnerabilities, but do not assume that vulnerability is a child’s ‘master identity’ (Christensen 2000, Prout 2005), fresh investigations of children’s encounters with neuroscience, and with biomedical technologies more generally, can critically inform deliberations on how to maximise the benefits and minimise the harm of children’s interactions with new technologies.

## Footnotes

1 The VOICES study was funded by the Wellcome Trust, grant no. 080209.

2 Interviews were stratified by age prior to random sampling to ensure even coverage of the age range in the final sub-sample. This enabled an investigation of age effects in children’s discourse about the brain. No age effects were found in this sample; however, this may indicate a limitation of the sampling process or of the analysis. Younger children generally display a more concrete reasoning style than older children (this is also demonstrated in findings from the VOICES study that pertain to children’s accounts of the self on and off medication; see Singh 2012); therefore, one would expect children of different ages to have somewhat different conceptions of the relations among self, brain and behaviour.

3 There is a possibility that the necessary structural similarities in the topic guides for diagnosed and undiagnosed children could have biased the undiagnosed sample toward less distinctive brain talk.

4 Most children we interviewed were not yet in the phase of adolescence when personal identity concerns strongly conflict with social norms for appropriate behaviour. I would expect more agitation about personal identity among older children being treated with stimulant medications.
